# Particulate Air Pollution, Metabolic Syndrome, and Heart Rate Variability: The Multi-Ethnic Study of Atherosclerosis (MESA)

**DOI:** 10.1289/ehp.0901778

**Published:** 2010-06-08

**Authors:** Sung Kyun Park, Amy H. Auchincloss, Marie S. O’Neill, Ronald Prineas, Juan C. Correa, Jerry Keeler, R. Graham Barr, Joel D. Kaufman, Ana V. Diez Roux

**Affiliations:** 1 Department of Environmental Health Sciences, School of Public Health, University of Michigan, Ann Arbor, Michigan, USA; 2 Department of Epidemiology and Biostatistics, School of Public Health, Drexel University, Philadelphia, Pennsylvania, USA; 3 Department of Epidemiology, School of Public Health, University of Michigan, Ann Arbor, Michigan, USA; 4 Division of Public Health Sciences, Wake Forest University School of Medicine, Winston-Salem, North Carolina, USA; 5 Fundacion Santa Fe de Bogota, Salud Comunitaria, Bogota, Colombia; 6 Department of Medicine and; 7 Department of Epidemiology, Columbia University Medical Center, New York, New York, USA; 8 Department of Environmental and Occupational Health Sciences, School of Public Health, University of Washington, Seattle, Washington, USA

**Keywords:** air pollution, autonomic nervous system, heart rate variability, metabolic syndrome, PM_2.5_

## Abstract

**Background:**

Cardiac autonomic dysfunction has been suggested as a possible biologic pathway for the association between fine particulate matter ≤ 2.5 μm in diameter (PM_2.5_) and cardiovascular disease (CVD). We examined the associations of PM_2.5_ with heart rate variability, a marker of autonomic function, and whether metabolic syndrome (MetS) modified these associations.

**Methods:**

We used data from the Multi-Ethnic Study of Atherosclerosis to measure the standard deviation of normal-to-normal intervals (SDNN) and the root mean square of successive differences (rMSSD) of 5,465 participants 45–84 years old who were free of CVD at the baseline examination (2000–2002). Data from the U.S. regulatory monitor network were used to estimate ambient PM_2.5_ concentrations at the participants’ residences. MetS was defined as having three or more of the following criteria: abdominal obesity, hypertriglyceridemia, low high-density lipoprotein cholesterol, high blood pressure, and high fasting glucose.

**Results:**

After controlling for confounders, we found that an interquartile range (IQR) increase in 2-day average PM_2.5_ (10.2 μg/m^3^) was associated with a 2.1% decrease in rMSSD [95% confidence interval (CI), −4.2 to 0.0] and nonsignificantly associated with a 1.8% decrease in SDNN (95% CI, −3.7 to 0.1). Associations were stronger among individuals with MetS than among those without MetS: an IQR elevation in 2-day PM_2.5_ was associated with a 6.2% decrease in rMSSD (95% CI, −9.4 to −2.9) among participants with MetS, whereas almost no change was found among participants without MetS (*p*-interaction *=* 0.005). Similar effect modification was observed in SDNN (*p*-interaction *=* 0.011).

**Conclusion:**

These findings suggest that autonomic dysfunction may be a mechanism through which PM exposure affects cardiovascular risk, especially among persons with MetS.

Over the past decades, numerous epidemiologic studies have shown an association of cardiovascular disease (CVD) with particulate air pollution exposures (Laden et al. 2006; Puett et al. 2008). Possible biologic pathways for this association include alterations in cardiac autonomic function, the release of circulating prooxidative and proinflammatory mediators from the lungs into the systemic circulation after particle inhalation, and translocation of ultrafine particles and soluble constituents of particles into the systemic circulation (Brook 2008). The pathway by which exposure to particulate pollutants adversely affects the cardiac autonomic nervous system may, in part, account for an increased risk of arrhythmias and sudden cardiac death associated with particulate exposure. In recent experimental studies, scientists prevented particle-induced cardiac oxidative stress by pretreating rats with autonomic nerve receptor antagonists (Rhoden et al. 2005) and particle-induced neuroplasticity of cardiac vagal neurons (Pham et al. 2009). The results of these studies have suggested that cardiac particle toxicity is mediated by autonomic nervous system stimulation.

Numerous epidemiologic studies have shown an association between particulate air pollution and cardiac autonomic dysfunction, as measured by heart rate variability (HRV) (Chuang et al. 2007; Holguin et al. 2003; Liao et al. 2004; Park et al. 2005; Whitsel et al. 2009). However, most previous studies have used a small number of subjects; only a few studies have been conducted in population-based cohorts (Liao et al. 2004; Park et al. 2005; Whitsel et al. 2009). Only one study (Park et al. 2005) examined an association with particulate matter (PM) ≤ 2.5 μm in aerodynamic diameter (PM_2.5_). Churg and Brauer (2000) have suggested that PM_2.5_ is more harmful to health than are larger particles [which are included in measurements of PM ≤ 10 μm in aerodynamic diameter (PM_10_)] because PM_2.5_ can penetrate deeper into the lung than PM_10_.

Some studies have found that associations between PM exposure and autonomic dysfunction are stronger among persons with type 2 diabetes (Park et al. 2005; Whitsel et al. 2009), hypertension (Holguin et al. 2003; Park et al. 2005), and obesity (Chen et al. 2007; Schwartz et al. 2005). The findings from these studies indicate that preexisting cardiometabolic diseases may confer susceptibility to cardiac autonomic dysfunction by particle exposure. Metabolic syndrome (MetS), a cluster of health risks characterized by abdominal obesity, type 2 diabetes, hypertension, and dyslipidemia (Moller and Kaufman 2005), has been associated with increased activity of the sympathetic nervous system, as have the individual conditions that define MetS (Tentolouris et al. 2008). In a recent study from Korea, Min et al. (2009) observed a significant reduction in HRV related to exposure to carbon monoxide (CO) among persons with MetS but not among persons without MetS (Min et al. 2009). Because of the increasing prevalence of MetS and its association with an increased risk of CVD, it is important to determine whether MetS or its individual components may increase susceptibility to the cardiovascular effects of air pollution.

We used data from the Multi-Ethnic Study of Atherosclerosis (MESA), a large population-based study, to investigate associations between HRV and PM_2.5_ and to assess potential effect modification by MetS and its components.

## Materials and Methods

### Study population

The MESA is a longitudinal study supported by the National Heart, Lung, and Blood Institute; its aim is to identify risk factors for subclinical atherosclerosis. A total of 6,814 participants 45–84 years old who were free of clinically apparent CVD at the baseline examination were recruited from six U.S. communities: Baltimore City and Baltimore County, Maryland; Chicago, Illinois; Forsyth County, North Carolina; Los Angeles County, California; northern Manhattan and the Bronx, New York; and St. Paul, Minnesota. Details of the study design are described elsewhere (Bild et al. 2002). Our study used data from the baseline visit that occurred between July 2000 and August 2002. Of the 6,814 participants who completed the baseline clinical examination, a total of 1,349 were excluded from the study because of nonparticipation in the air pollution study (*n* = 618), missing or poor quality electrocardiogram (ECG) data (*n* = 401), missing information on PM_2.5_ exposure (*n* = 160), missing information on both ECG and PM_2.5_ (*n* = 65), or missing information on temperature (*n* = 11) and other covariates (*n* = 94). Therefore, 5,465 participants with complete data were available for the analyses. Compared with other participants, those who were excluded (*n* = 1,349) were older (65 years old vs. 62 years old), more likely to be African American (33% vs. 27%) and current smokers (16% vs. 12%), more likely to be from Baltimore (21% vs. 15%) and less likely to be from Chicago (13% vs. 18%), and at higher risk of hypertension (22% vs. 18%) and type 2 diabetes (25% vs. 19%). The study protocol was approved by the institutional review board at each study site, and written informed consent was obtained from each participant.

### Air pollution and meteorologic data

Air pollutant data were extracted from the U.S. Environmental Protection Agency (EPA) Aerometric Information Retrieval System (U.S. EPA 2007). PM_2.5_ concentrations were obtained from 24-hr integrated samplers, which collected data daily or every third day. For each participant, we obtained PM_2.5_ concentration data for the 60 days before the day on which HRV measures were obtained. Each daily exposure measure was obtained from the monitor nearest to the participant’s residence with available data for a given day. We constructed five exposure measures: PM_2.5_ concentration the day before HRV measurement, and average PM_2.5_ concentrations over the 2, 7, 30, and 60 days prior to HRV measurement. Gaseous pollutants [sulfur dioxide (SO_2_), nitrogen dioxide (NO_2_), and CO] were also included. Ozone was not analyzed because of incomplete information in the winter. Meteorologic data including temperature and dew point temperature were obtained from the National Climatic Data Center (NCDC 2008). To control for weather, we used apparent temperature, defined as a person’s perceived air temperature, which combines the effects of heat and humidity in one variable (O’Neill et al. 2003).

### HRV measurement

After at least a 5-min rest, three consecutive 10-sec recordings of simultaneous 12-lead ECGs (Marquette MAC-1200; GE Healthcare, Milwaukee, WI, USA) were measured and read electronically after transmission over analog phone lines to a central ECG reading center that was blinded to all clinical and personal details of the participants. Most (92%) of the ECGs were assessed in the morning. ECG abnormalities were identified by a program using Nova code criteria (Rautaharju et al. 1998) and Minnesota code criteria (Prineas et al. 1982). On ECGs with > 50% normal-to-normal (NN) interbeat intervals, two ECG measures of HRV were calculated using three consecutive 10-sec recordings: the standard deviation of NN intervals (SDNN) and the root mean square of successive differences of NN intervals (rMSSD). In general, SDNN and rMSSD measures based on ultrashort (e.g., 10 sec) recordings indicate short-term, resting parasympathetic (respiratory) variation. We excluded participants who had only one 10-sec HRV measure (1%); averages of two (7%) or three (92%) 10-sec HRV measures were computed and included in the statistical analysis. The validity of the HRV calculation method was verified by senior electrocardiographers and a biostatistician using a test data set of 264 ECGs. Prior work has shown high correlations between 10-sec and 6-min measures: correlation coefficients [95% confidence intervals (CIs)] 0.76 (0.68–0.82) for SDNN and 0.82 (0.75–0.86) for rMSSD (Schroeder et al. 2004).

### MetS and individual metabolic abnormalities

We used the criteria of the National Cholesterol Education Program Adult Treatment Panel III (Moller and Kaufman 2005) and defined persons with three or more of the following criteria as having MetS: *a*) abdominal obesity (waist circumference > 102 cm in men or > 88 cm in women); *b*) hypertriglyceridemia (≥ 150 mg/dL); *c*) low high-density lipoprotein (HDL) cholesterol (< 40 mg/dL in men or < 50 mg/dL in women); *d*) high blood pressure (≥ 130/85 mm Hg or use of antihypertensive medication); and *e*) impaired fasting glucose (≥ 110 mg/dL, or self-reported diabetes or use of diabetes medication). We used clinical cutoff points for each of the individual components of MetS rather than the criteria used in MetS above to maintain a high degree of specificity for each disease. Diabetes was defined as fasting blood glucose of ≥ 126 mg/dL and/or use of diabetes medication (insulin or oral hypoglycemic agents). Hypertension was defined as blood pressure ≥ 140/90 mm Hg or use of hypertension medication. Dyslipidemia was defined as having both hypertriglyceridemia and low HDL cholesterol.

### Statistical analysis

Linear regression analyses were used to test for the associations between HRV measures and PM_2.5_. HRV measures were log transformed to normalize and to stabilize the variance. The following potential confounding variables were selected *a priori* and included in the model regardless of statistical significance: age, sex, race/ethnicity, cigarette smoking, body mass index (BMI), fasting blood glucose, mean arterial pressure, use of cardiac medications (beta blockers, angiotensin-converting enzyme inhibitors, and calcium channel blockers), and the lagged moving average of apparent temperature corresponding to the same moving average period for PM_2.5_. Categorical variables were modeled as shown in Table 1. We used beta blockers, angiotensin-converting enzyme inhibitors and calcium channel blockers as classes of antihypertensive medications to make our models comparable with the models used in Park et al. (2005). The results did not change when we used an indicator variable for all possible antihypertensive medications, including diuretics and angiotensin II receptor antagonists. We also considered education, alcohol consumption, physical activity, serum total cholesterol, serum triglyceride, and use of statins in our models, but only serum triglyceride was included in final models, as it was the only one that changed the beta coefficients of PM_2.5_ by > 10%. To account for the nonlinear association of apparent temperature with HRV, we used a regression spline with 3 degrees of freedom (df). Season may be an important confounder, because both PM_2.5_ concentrations and HRV show seasonal variations. In MESA, seasons vary by study sites; for example, winter in Los Angeles is different from winter in Chicago or New York. To control for seasonality at different study sites, we added interaction terms between splines of apparent temperature (3 df) and study sites. This model captures site-specific seasonality. We also created an indicator variable of warm months by study sites. Warm months were defined as those in which the average monthly temperature was greater > 10°C (50°F). For Baltimore, Forsyth County, and New York, warm months occurred from April to October. For Chicago and St. Paul, they occurred from May to October. Los Angeles has warm months all year long. We added this indicator variable to the model to remove any residual confounding by season. We also examined possible confounding effects of gaseous pollutants (SO_2_, NO_2_, and CO) and their independent associations with HRV.

We checked for possible nonlinear relations between HRV and other continuous covariates using penalized splines, but there was no evidence of strong nonlinear relations; thus, we fit all other continuous covariates as linear terms. We present effect estimates as percent change for an interquartile range (IQR) increase for PM_2.5_. We checked whether the relation between PM_2.5_ and HRV was linear by fitting PM_2.5_ as a smoothing term using penalized splines. This smoothing method makes no assumptions regarding the shape of the association (Wood 2006), and the penalized splines can be estimated in a generalized additive model using R (version 2.9.2; The R Foundation for Statistical Computing, Boston, MA).

Study site may be an important confounder or an effect modifier, because the composition of PM_2.5_ may vary by different locations. Two analytical approaches were used to account for site: one was to additionally adjust for study site in the regression model as described above, and the other was to construct site-specific regression models and pool the estimates using meta-analysis. Heterogeneity of effect estimates was assessed with a χ^2^ test with 5 df. If heterogeneity by study sites was found, random-effect models were used to obtain a pooled estimate while accounting for site heterogeneity.

To assess effect modification by MetS and its individual components, multiplicative interaction terms along with the main effects were included in regression models. We also examined effect modification by other individual-level factors, including sex, education, cigarette smoking, cardiac medication, and use of statins. The *p*-value of significance was < 0.05.

## Results

Table 1 shows the distribution of demographic and clinical characteristics of the study participants. The average age of participants was 62 years, and approximately half of the participants were female. The prevalence of hypertension, type 2 diabetes, abdominal obesity, dyslipidemia, and MetS was 43%, 12%, 54%, 19%, and 32%, respectively. Persons with MetS were older, more likely to be women, and to have had lower HRV measures. Two days before the HRV measurement, PM_2.5_ concentrations averaged from 10.3 μg/m^3^ in St. Paul to 19.3 μg/m^3^ in Los Angeles, with an overall mean of 14.3 μg/m^3^. Overall, the correlations between PM_2.5_ and the corresponding moving averages of apparent temperature were modest (Spearman correlation coefficients between 0.21 and 0.31). Month averages of PM_2.5_ concentrations (30-day and 60-day means for PM_2.5_) were moderately correlated with shorter-term averages (prior day and 2-day mean PM_2.5_) (Spearman correlation coefficients 0.35 to 0.48).

Table 2 shows adjusted geometric means and geometric standard errors of HRV measures for sex, race/ethnicity, and study sites, stratified by MetS. In general, persons with MetS had lower HRV values than those without MetS. Women and African Americans had higher HRV measures than men and other race/ethnic groups, respectively. HRV measures differed by the study sites. For persons without MetS, HRV measures were lower in Baltimore and Chicago and higher in Forsyth County, Los Angeles, and St. Paul. For persons with MetS, Baltimore and Forsyth County had higher values, whereas Los Angeles had the lowest values. The differences in HRV values between persons with and without MetS were greatest in Los Angeles, and no differences in MetS status were observed among participants in Baltimore.

Table 3 presents adjusted associations of HRV measures with various lags of PM_2.5_. All PM_2.5_ lags showed inverse associations with HRV measures, and the associations were generally strongest for the 2-day moving average, especially for SDNN. Model 1 included age, sex, race/ethnicity, smoking status, BMI, fasting blood glucose, mean arterial pressure, serum triglyceride, cardiac medications, indicator of warm months, and regression splines of apparent temperature (3 df) as covariates. Model 2 added interaction terms between study sites and regression splines of apparent temperature along with main terms for sites and apparent temperature. This additional adjustment for site-specific apparent temperature made the associations slightly stronger for shorter moving averages of PM_2.5_ (1-day previous and 2-day and 7-day averages), whereas the associations with longer moving averages (30-day and 60-day averages) became weaker after such an adjustment. In Model 2, one IQR increment in 2-day average of PM_2.5_ (10.2 μg/m^3^) was associated with decreases of 1.84% (95% CI, −3.73 to 0.08) and 2.10% (95% CI, 0.0 to 4.17) in SDNN and rMSSD, respectively. Associations became stronger after adjustment for gaseous copollutants in all lag models (Model 3), with more pronounced changes noted when NO_2_ or CO were added to models (data not shown). There were no significant associations of HRV with gaseous pollutants (data not shown).

We conducted regression analyses for the 2-day moving average stratified by study site and controlled for covariates used in Model 1 to check heterogeneity of site-specific associations. No significant heterogeneity of site-specific associations was found (*p*-values for heterogeneity = 0.27 for SDNN and 0.11 for rMSSD). Overall associations from the meta-analysis were −1.83% (95% CI, −3.71 to 0.08) for SDNN and −2.03% (95% CI, −4.08 to 0.07) for rMSSD (data not shown).

**Figure 1:**
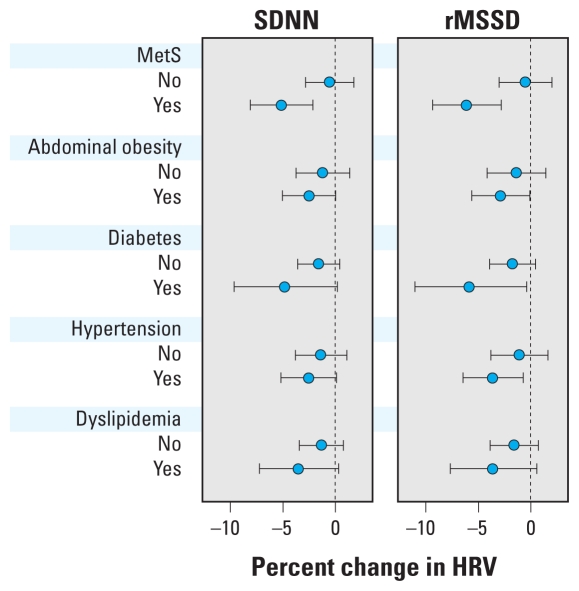
Adjusted percent changes and 95% CIs in HRV measures for an IQR increase in PM_2.5_ (10.2 μg/m^3^) by MetS and its components (abdominal obesity, diabetes, hypertension, and dyslipidemia), MESA, 2000–2002. The adjusted covariates are the same as in Model 2, Table 3, except for the covariates that are part of each effect modifier: The model for MetS did not include BMI, fasting glucose, mean arterial pressure (MAP), serum triglyceride, and cardiac medications (beta blockers, calcium channel blockers, ACE inhibitors); the model for abdominal obesity did not include BMI; the model for diabetes did not include fasting glucose; the model for hypertension did not include mean arterial pressure and cardiac medications; and the model for dyslipidemia did not include serum triglyceride.

Figure 1 shows results for effect modification by MetS and its individual components in association between HRV and 2-day average of PM_2.5_. We found statistically significant effect modification by MetS. One IQR increase in 2-day PM_2.5_ was significantly associated with 5.16% (95% CI, 2.13–8.10) and 6.16% (95% CI, 2.85–9.35) decreases in SDNN and rMSSD, respectively, among persons with MetS, whereas no relationship was found among persons without MetS (*p*-values for interaction = 0.011 for SDNN and 0.005 for rMSSD). We also found significant associations of PM_2.5_ and rMSSD among persons with abdominal obesity, diabetes, or hypertension, but no significant associations were observed among persons without those conditions. The interaction terms between PM_2.5_ and each individual component of MetS in each model were not statistically significant. We also examined effect modification by sex, education, cigarette smoking, cardiac medication (beta blockers, angiotensin-converting enzyme inhibitors, and calcium channel blockers), and statin use, but no significant effect modification was observed (data not shown).

## Discussion

This cross-sectional, multiethnic, multicity study showed that exposure to greater levels of fine particles averaged for 2 days before the HRV measurement was associated with less HRV, as indicated by lower short-term recorded SDNN and rMSSD, markers of resting parasympathetic (vagal) variation of the heart. Individuals with MetS had significantly larger decreases in HRV measures in relation to PM_2.5_ than did those without MetS. The associations were also consistently stronger among persons with individual components of MetS (such as abdominal obesity, diabetes, hypertension, or dyslipidemia) than among persons without the components, although the differences were not statistically significant. These findings suggest that possible cardiovascular effects of PM_2.5_ may be mediated, in part, through altered autonomic function, particularly among persons with MetS. The observed associations did not differ by sex, race/ethnicity, study site, educational attainment, smoking status, and use of antihypertension and anticholesterol medications. These results were independent of individual-level confounders as well as other environmental factors.

Our findings are consistent with the results from a previous community-based study of HRV and PM_2.5_, but the magnitude of the associations in our study is smaller. A study conducted with 497 elderly men from the Normative Aging Study (NAS) found that the association of HRV was strongest with the 2-day (48-hr) average of PM_2.5_ (Park et al. 2005). In the present study, we modeled the same covariates used in the NAS to facilitate comparisons between the studies. In the NAS population, 8 μg/m^3^ of PM_2.5_ was associated with a 5.4% (95% CI, −1.5 to 11.8) decrease in SDNN, which is approximately three times larger than the magnitude of the association in our study. NAS participants with hypertension or diabetes were also more susceptible to PM_2.5_ exposure than were the participants without these conditions. Other population-based studies have examined PM_10_ rather than PM_2.5_. Liao and colleagues examined 4,899 middle-aged and elderly participants from the Atherosclerosis Risk in Communities Study and found that 11.5 μg/m^3^ of PM_10_ measured 1-day before was associated with a 1.03-msec (SE = 0.31) reduction in SDNN, which is equivalent to a 2.75% (95% CI, 1.13–4.37) decrease (based on the population mean of SDNN = 37.5 msec) (Liao et al. 2004). They also found a stronger association among persons with hypertension.

Our study supports previous evidence that preexisting cardiometabolic diseases may confer susceptibility to particle-induced autonomic dysfunction. Furthermore, only MetS was identified as a significant modifying factor, and interactions between PM_2.5_ and its individual components did not reach statistical significance. This suggests that a clustering of cardiometabolic risk factors increases susceptibility to the adverse effects of particle exposure. MetS has been associated with diminished HRV among middle-aged and elderly persons (Gehi et al. 2009; Liao et al. 1998; Stein et al. 2007) and even young adults (Koskinen et al. 2009). Persons with MetS are characterized by central fat accumulation, insulin resistance, glucose intolerance, and increased inflammation, which are associated with an overactivation of the sympathetic nervous system (Tentolouris et al. 2008). Sympathetic overactivity may have unfavorable impacts on the cardiovascular system such as development of hypertension, endothelial dysfunction, and heart failure (Tentolouris et al. 2006). It is unclear why and how MetS modifies the cardiovascular impact of particle exposure. We can speculate that particle exposure worsens the autonomic dysfunction caused by overactivation of the sympathetic nervous system because of MetS, but further toxicologic studies are needed to elucidate the biologic role of MetS in the association between particle exposure and autonomic function.

This study was conducted in six different cities in the United States with a uniform protocol including questionnaires, physical measurements, and analyses of laboratory and ECG measurements in centralized core laboratories. However, different cities have samples with different sociodemographic characteristics; they differ in meteorological conditions and also vary substantially in PM_2.5_ exposures. To account for this heterogeneity, we not only conducted regression analyses adjusting for study site and site-specific apparent temperature as smoothing terms, but we conducted meta-analysis using site-specific regression models. The observed associations were similar in analyses that adjusted for site as a covariate and in the meta-analysis approach.

The associations between PM_2.5_ and HRV measures became much stronger after adjustment for gaseous co-pollutants, especially NO_2_ and CO. Recent studies conducted in the United States suggest that ambient gaseous pollutant concentrations (such as CO and NO_2_) determined from central monitoring stations may not be good surrogates for corresponding individual-level gaseous exposures in epidemiologic research (Sarnat et al. 2005; Schwartz et al. 2007). Zeger and colleagues suggested that the effect estimate can be biased away from the true value if there is a strong negative correlation between measurement errors, because some of the effect of a more poorly measured variable may be transferred to the estimate of a better-measured variable (Zeger et al. 2000). Given that measurement errors for ambient gaseous pollutant are greater than those for ambient particles, adjustment for gaseous pollutants may lead to an overestimation of the true effect of particles.

Although statistically significant associations between PM_2.5_ and HRV measures were seen in this study, the magnitude of the associations was relatively small: approximately 2% decreases in SDNN and rMSSD with a 10.2-μg/m^3^ increase in 2-day average PM_2.5_ (Model 2, Table 3). These decrements were roughly equivalent to a cross-sectional reduction in HRV associated with about 2 years of aging in our sample. Given the ubiquitous nature of particle exposure and the dose-dependent relation between reduced HRV and incidence of cardiovascular events, even those small effects, if causal, have the potential to influence public health.

An important strength of our study is that the analysis was based on a large community sample that included both sexes and various ethnic groups and cities, with a large number of participants. However, several limitations should be considered. MESA included a relatively healthy sample of persons without a prior history of CVD. If prior disease modifies the effects of PM on autonomic function, our results may not be generalizable to samples with preexisting cardiovascular conditions. However, the investigation of associations in large healthy samples is still of interest. The use of a healthy sample also minimizes residual confounding due to health conditions that could be related to HRV and to PM exposure, if persons with pre-existing conditions tend to live in or move to areas with greater PM exposure. Additionally, we restricted the analysis to participants with complete values for the variables included in the regression models. This approach may lead to bias if the missing values are not completely random (Raghunathan 2004). Subjects included in the analysis were relatively healthier than those excluded, and there were small differences in exclusion rates by sites. Although we cannot categorically rule out selection bias, it is unlikely that subjects were systematically excluded based on outcome (i.e., HRV) and exposure data. MESA study sites use the same standardized protocols for quality control; hence, systematic differences in HRV measurement between sites or across subjects with different characteristics are unlikely. Although multiple imputation of missing data is an option, it requires a number of assumptions. Given the approach taken in prior MESA analyses (which have not used multiple imputation), we report results based on participants with complete data.

We assessed HRV using three consecutive 10-sec recordings. Generally, use of records at least 5 min in duration is recommended in accordance with the previous guidelines (Task Force 1996). However, there has been interest in using ultrashort recordings, such as 10 sec in duration, from the standard 12-lead ECG, because these records are much easier to collect in clinical and epidemiologic settings (de Bruyne et al. 1999; Dekker et al. 1997). It has been suggested that repeatability of HRV measures from 10-sec recordings is high when the mean from two or three records is used, and correlations between the same measures from 10 sec and 6 min were quite high (Schroeder et al. 2004). In our study, we recorded 10-sec ECGs three times and excluded participants with one 10-sec record. Because SDNN reflects overall variability of heart rate, which requires a fair amount of time to be computed (generally longer than 5 min), the physiologic meaning of SDNN (overall variability) measured for 10 sec may capture only part of SDNN derived from longer periods (de Bruyne et al. 1999). On the other hand, rMSSD reflects short-term variability seen with respiration at rest and is thought to be a marker of the parasympathetic nervous system variation (Task Force 1996). Therefore, the 10-sec SDNN and rMSSD measures used in this study might reflect the same phenomenon, that is, parasympathetic modulation of the heart. The ultrashort records did not allow evaluation of frequency domain.

Another limitation is single measurement of HRV because of the cross-sectional design, which cannot rule out residual confounding by subject-specific characteristics. Previous studies from the NAS showed consistent findings in both cross-sectional (Park et al. 2005) and longitudinal designs (Baccarelli et al. 2008), and the magnitude of the associations did not change. Nevertheless, the future availability of repeated HRV measurements in this sample will allow analysis that relates within-person changes in HRV to within-person changes in exposures, thus tightly accounting for subject-specific confounders.

Like many other population-based air pollution studies, we did not measure person-level pollution exposure. We used PM_2.5_ exposure from the monitor nearest each participant’s residence. This method is likely an improvement over using a single representative monitor located in the center of a city. In MESA, 38% of participants were not employed at the time of the survey (retired and not working, unemployed, or homemakers), and more than 75% reported spending 60% of their time either in their home or within 1 mile (1.6 km) of their home. This suggests that the exposure assignment based on participant’s residence used in this study is reasonable. Nevertheless, we cannot exclude a possibility of exposure misclassification; however, such an error is likely to be nondifferential.

In summary, short-term exposure to PM_2.5_ was marginally significantly associated with cardiac autonomic dysfunction, as manifested by decreased HRV measures in a large, multicity, multi-ethnic, population-based sample. In addition, persons with MetS showed a larger reduction in HRV associated with PM exposures, suggesting MetS as a susceptibility factor to particle-induced autonomic dysfunction. Given the potential limitations, these results must be interpreted with caution. Our findings support the hypothesis that alterations in cardiac autonomic function may be one of the underlying mechanisms through which particulate air pollution increases the risk of CVD. The biologic role of MetS in enhancing susceptibility to particle toxicity remains to be determined.

## Figures and Tables

**Table 1:** Demographic and clinical characteristics of the study population with complete data, MESA 2000–2002.

		MetS	
Characteristic	Total (*n* = 5,465)	No (*n* = 3,698)	Yes (*n* = 1,767)
Age (years)	62.2 ± 10.9	61.5 ± 10.2	63.5 ± 9.8
Percent female	53.2	51.0	57.8
Race/ethnicity (%)			
African American	26.6	26.8	26.0
Caucasian	38.9	40.4	35.8
Chinese	12.4	13.7	9.6
Hispanic	22.2	19.1	28.6
Education (%)			
≤ High school diploma	34.9	31.2	42.5
Some college	28.8	28.1	30.2
≥ 4-year college	36.3	40.6	27.3
Study site (%)			
Baltimore	14.7	14.7	14.8
Chicago	18.1	20.4	13.1
Forsyth County	15.6	14.9	17.0
Los Angeles	19.8	19.2	21.0
New York	16.1	16.6	15.0
St. Paul	15.8	14.2	19.1
Smoking status (%)			
Never	51.0	51.1	50.9
Former	36.6	36.8	36.1
Current	12.4	12.1	13.0
Hypertension (%)	43.4	32.3	66.8
Diabetes (%)	11.8	3.8	28.6
Abdominal obesity (%)	53.7	38.6	85.2
Dyslipidemia (%)	18.7	3.8	49.8
Use of hypertension medication (%)	35.9	25.6	57.5
Beta blockers (%)	8.5	5.6	14.4
Ca^2+^ channel blockers (%)	11.7	8.8	17.7
ACE inhibitors (%)	11.3	6.7	20.8
BMI (kg/m^2)^	28.3 ± 5.4	27.0 ± 4.9	31.1 ± 5.2
Waist circumference (cm)	97.9 ± 14.2	94.0 ± 13.3	106.0 ± 12.5
Fasting glucose (mg/dL)	96.5 ± 28.5	89.9 ± 17.6	110.4 ± 39.7
Mean arterial pressure (mmHg)	89.8 ± 12.3	88.2 ± 12.1	93.2 ± 12.1
HDL (mg/dL)	51.0 ± 14.8	54.9 ± 14.9	42.8 ± 10.5
Triglyceride (mg/dL)	131.9 ± 88.3	105.6 ± 58.2	186.8 ± 112.1
Heart rate (beats/min)	63.0 ± 9.4	62.0 ± 9.0	65.1 ± 10.0
HRV, GM (GSD)			
SDNN (msec)	18.8 (1.84)	19.5 (1.81)	17.4 (1.91)
rMSSD (msec^2^)	20.9 (1.96)	21.7 (1.91)	19.4 (2.04)
Environmental variables, [prior 2 days, median (IQR)]			
PM_2.5_ (μg/m^3^)	14.3 (10.2–20.4)	14.4 (10.2–20.4)	14.0 (10.1–20.4)
NO_2_ (ppb)	23.5 (15.8–31.5)	23.9 (16.5–31.9)	22.6 (15.3–30.9)
SO_2_ (ppb)	3.6 (1.8–6.6)	3.7 (1.8–6.8)	3.3 (1.6–6.1)
CO (ppm)	0.90 (0.65–1.35)	0.90 (0.70–1.35)	0.90 (0.65–1.30)
Apparent temperature (°C)	11.5 (3.5–17.5)	11.3 (3.1–17.5)	11.6 (4.0–17.3)

Abbreviations: GM, geometric mean; GSD, geometric standard deviation. Unless otherwise noted, values are reported as mean ± SD.

**Table 2:** Adjusted HRV for sex, race/ethnicity, and study sites by MetS, MESA, 2000–2002.

	MetS					
	No (*n* = 3,698)			Yes (*n* = 1,767)		
Characteristic	*n*	SDNN, msec	rMSSD, msec	*n*	SDNN, msec	rMSSD, msec
Sex						
Women	1,884	19.47 (1.01)	22.54 (1.02)	1,022	17.90 (1.02)	20.33 (1.02)
Men	1,814	18.99 (1.01)	20.76 (1.02)	745	16.63 (1.03)	18.16 (1.03)

Race/ethnicity						
African American	991	22.16 (1.02)	26.14 (1.02)	460	20.05 (1.03)	23.72 (1.04)
Caucasian	1,494	19.32 (1.02)	20.28 (1.02)	632	17.10 (1.03)	18.04 (1.03)
Chinese	505	17.19 (1.03)	19.56 (1.03)	170	15.87 (1.06	17.93 (1.06
Hispanic	708	18.57 (1.02)	21.12 (1.03)	505	16.29 (1.03)	17.76 (1.04)

Study site						
Baltimore	542	18.14 (1.03)	20.41 (1.03)	262	18.62 (1.05)	20.78 (1.05)
Chicago	755	18.14 (1.02)	20.31 (1.02)	232	17.35 (1.04)	18.41 (1.05)
Forsyth County	551	20.54 (1.03)	22.18 (1.03)	300	18.04 (1.04)	19.88 (1.05)
Los Angeles	709	20.10 (1.02)	22.49 (1.02)	371	15.78 (1.04)	17.53 (1.04)
New York	615	18.54 (1.02)	21.30 (1.03)	265	17.17 (1.04)	19.53 (1.05)
St. Paul	526	20.08 (1.03)	23.28 (1.03)	337	16.72 (1.04)	19.33 (1.04)

Measures were log transformed for analysis and then exponentiated. Values are geometric means (geometric standard errors) adjusted for age and for the other two variables in the table.

**Table 3:** Adjusted percent change (95% CI) in HRV measures for an IQR increase in PM_2.5_ in models with various lagged exposure measures, MESA 2000–2002.

	SDNN		rMSSD	
Model	Percent change (95% CI)	*p*-Value	Percent change (95% CI)	*p*-Value
1-day previous (IQR = 11.45 μg/m^3^)				
Model 1	−1.18 (−2.92 to 0.59)	0.19	−1.67 (−3.57 to 0.26)	0.09
Model 2	−1.49 (−3.35 to 0.40)	0.12	−1.82 (−3.85 to 0.25)	0.08
Model 3	−1.67 (−3.77 to 0.47)	0.13	−2.02 (−4.32 to 0.32)	0.09

2-day moving average (IQR = 10.20 μg/m^3^)				
Model 1	−1.57 (−3.32 to 0.20)	0.08	−1.99 (−3.89 to −0.05)	0.04
Model 2	−1.84 (−3.73 to 0.08)	0.06	−2.10 (−4.17 to 0.00)	0.05
Model 3	−2.25 (−4.38 to −0.08)	0.04	−2.61 (−4.93 to −0.23)	0.03

7-day moving average (IQR = 7.66 μg/m^3^)				
Model 1	−0.53 (−2.32 to 1.29)	0.56	−1.04 (−2.99 to 0.95)	0.30
Model 2	−0.74 (−2.80 to 1.36)	0.49	−1.19 (−3.43 to 1.11)	0.31
Model 3	−0.83 (−3.17 to 1.57)	0.50	−1.49 (−4.04 to 1.12)	0.26

30-day moving average (IQR = 6.18 μg/m^3^)				
Model 1	−1.10 (−3.19 to 1.05)	0.31	−2.14 (−4.41 to 0.19)	0.07
Model 2	−0.69 (−3.57 to 2.28)	0.64	−1.77 (−4.90 to 1.46)	0.28
Model 3	−1.90 (−5.28 to 1.61)	0.28	−3.72 (−7.36 to 0.07)	0.05

60-day moving average (IQR = 5.32 μg/m^3^)				
Model 1	−0.87 (−3.04 to 1.36)	0.44	−1.79 (−4.16 to 0.64)	0.15
Model 2	−0.25 (−3.56 to 3.18)	0.89	−1.09 (−4.70 to 2.65)	0.56
Model 3	−1.81 (−5.80 to 2.35)	0.39	−3.74 (−8.03 to 0.76)	0.10

Model 1 adjusted for age, sex, race/ethnicity, smoking status, BMI, fasting glucose, mean arterial pressure, serum triglyceride, use of beta blockers, use of Ca^2+^ channel blockers, use of ACE inhibitors, season (warm vs. cold season), and regression splines (3 df) for moving averages of apparent temperature corresponding for the predictor. Model 2 includes Model 1 plus interactions between study sites and regression splines (3 df) for moving averages of apparent temperature corresponding for the predictor. Model 3 includes Model 2 plus gaseous copollutants (NO_2_, SO_2_, CO).
